# Flavonifractor plautii Bacteremia With Generalized Peritonitis: A Case Report and Literature Review

**DOI:** 10.7759/cureus.63890

**Published:** 2024-07-05

**Authors:** Shogo Saito, Shigeaki Baba, Haruka Nikai, Ryosuke Fujisawa, Tohru Fujiwara

**Affiliations:** 1 Division of Central Clinical Laboratory, Iwate Medical University Hospital, Yahaba, JPN; 2 Department of Surgery, Iwate Medical University School of Medicine, Yahaba, JPN; 3 Department of Laboratory Medicine and Infectious Diseases, Iwate Medical University School of Medicine, Yahaba, JPN

**Keywords:** piperacillin/tazobactam, maldi-tof ms, generalized peritonitis, bacteremia, flavonifractor plautii

## Abstract

*Flavonifractor plautii* is an obligate anaerobic rod bacterium that is part of the human gut microbiota. We describe a case of bacteremia caused by *F. plautii* in a mildly immunocompromised patient with acute generalized peritonitis. The patient is an 83-year-old male, with a history of stage III hepatocellular carcinoma 11 months prior, stage I gastric cancer, and cerebral infarction three months prior. He visited the emergency room of our hospital with a chief complaint of right-sided abdominal pain. A partial resection of the colon was performed due to stenosis of the transverse colon. Due to increasing abdominal pain, the patient underwent surgery for acute generalized peritonitis on the 11th postoperative day. *F. plautii* was detected in blood cultures collected prior to surgery, and the patient was treated with piperacillin/tazobactam 2.25 g four times a day for 11 days. The patient resumed eating and was discharged with no recurrence. This species may also stain gram-negative, and caution should be exercised in reporting results due to the potential impact on initial antimicrobial therapy. Gram staining showed variation in the length of the bacterium, which is considered a characteristic of this species. Appropriate antimicrobial therapy for *F. plautii* has yet to be established, and further accumulation of cases is needed to understand the resistance mechanism and confirm the effectiveness of different antimicrobials.

## Introduction

*Flavonifractor plautii* is an obligate anaerobic rod bacterium named in 2010 from the merger of *Eubacterium plautii* and *Clostridium orbiscindens*. This species is part of the human gut microbiota, belonging to the order *Eubacteriales*, family *Oscillospiraceae*, and is the type species of the genus *Flavonifractor* [1]. There have been few reports of infections caused by *F. plautii*, mostly in immunocompromised patients, and only one case reported in an immunocompetent patient. The clinical features of the infection remain largely unknown [2-7]. In this report, we describe a case of bacteremia caused by *F. plautii* in a mildly immunocompromised patient with acute generalized peritonitis.

## Case presentation

The patient is an 83-year-old male, is 161.2 cm tall, and weighs 53.1 kg, with underlying type 2 diabetes mellitus and hypertension and a history of stage III hepatocellular carcinoma with metabolic dysfunction-associated steatotic liver disease (MASLD) 11 months ago, stage I gastric cancer, and cerebral infarction three months ago. He has no allergies or significant family medical history. His diabetes is well controlled with metformin hydrochloride. Due to the recurrence of hepatocellular carcinoma, he underwent transcatheter arterial chemoembolization (TACE) and radiofrequency ablation (RFA) seven months ago. Three months prior, he had a laparoscopic distal gastrectomy (LDG) with a diagnosis of early gastric cancer (T1bN0M0) and was under follow-up.

**Figure 1:**
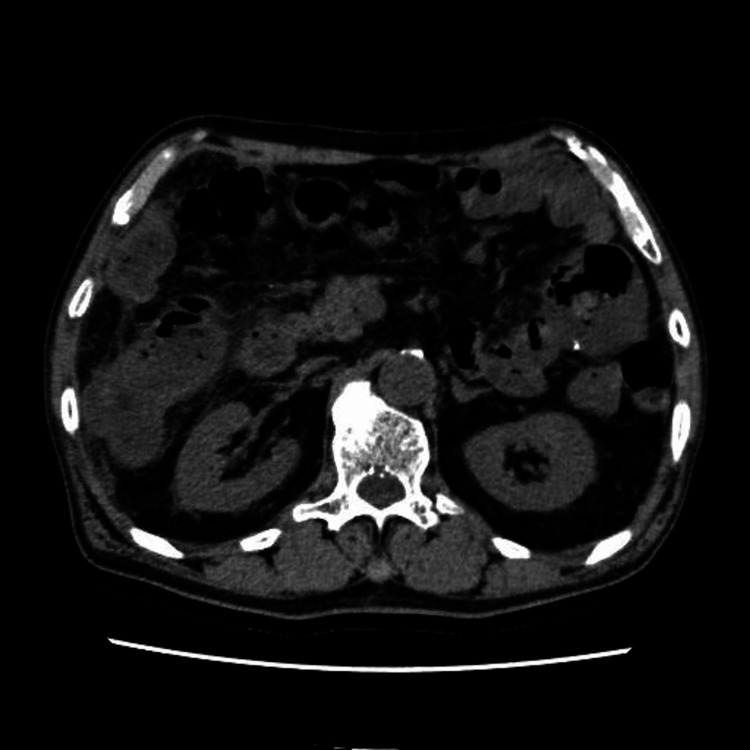
Computed tomography image of suspected bowel obstruction due to the thickening of the colon wall at the hepatic flexure.

**Figure 2:**
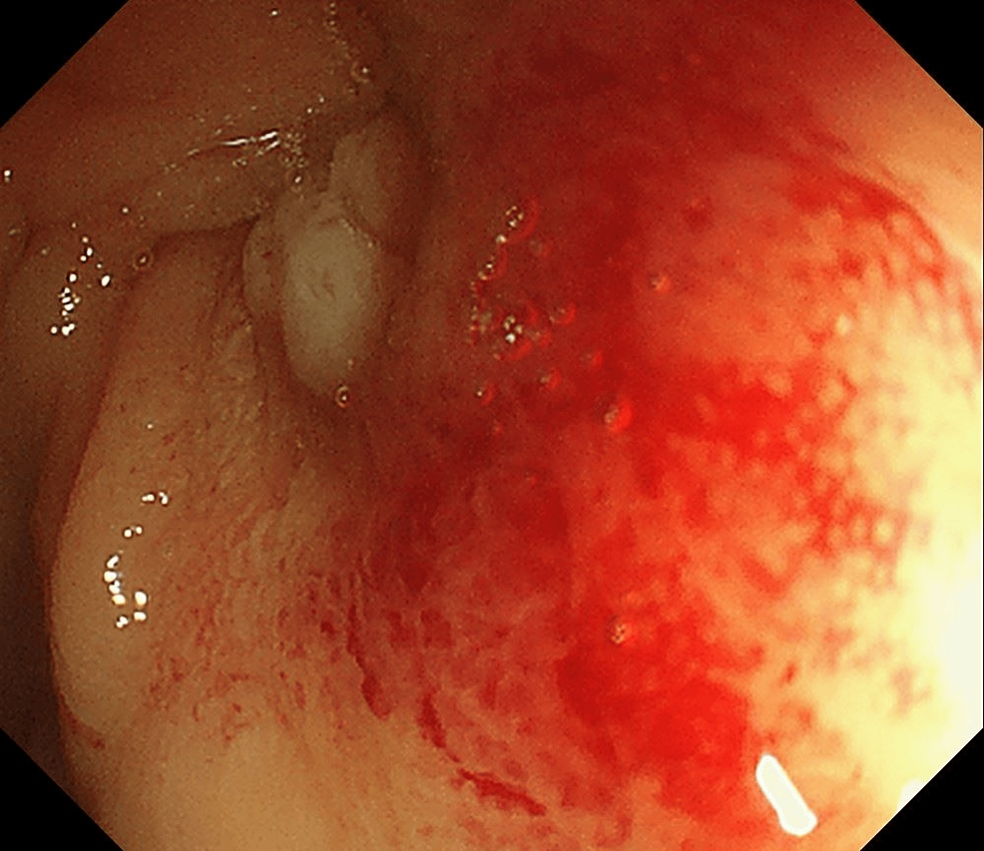
Lower gastrointestinal endoscopic image showing stenosis in the transverse colon.

After surgery, the patient had no recurrence and was leading a normal life until he visited the emergency room of our hospital with right-sided abdominal pain. He reported intermittent pain throughout the lower abdomen, with the right lower abdomen as the most tender point. Physical examination revealed clear consciousness, temperature 36.8°C, heart rate 66/min, blood pressure 143/77 mmHg, respiratory rate 14/min, and percutaneous oxygen saturation (SpO2) 96%. Laboratory results showed total protein 7.0 g/dL, albumin 3.7 g/dL, blood urea nitrogen 7.6 mmol/L, creatinine 113 µmol/L, C-reactive protein 1.6 mg/L, white blood cell 6.45 × 10^9^/L, red blood cell 3.24 × 10^12^/L, hemoglobin 104 g/L, platelet 254 × 10^9^/L, and D-dimer 2.7 µg/mL. Computed tomography revealed a suspected bowel obstruction due to the thickening of the colon wall at the hepatic flexure (Figure 1). A lower gastrointestinal endoscopy showed stenosis in the transverse colon where the scope could not pass (Figure 2). Partial resection of the colon was performed due to stenosis caused by adhesion between the previously performed TACE site and the greater omentum of the transverse colon. On postoperative day 7, he resumed eating, but by postoperative day 9, he developed abdominal pain, chills, vomiting, and hypotension. Due to worsening abdominal pain, he underwent surgery for acute generalized peritonitis on the 11th postoperative day.

**Figure 3:**
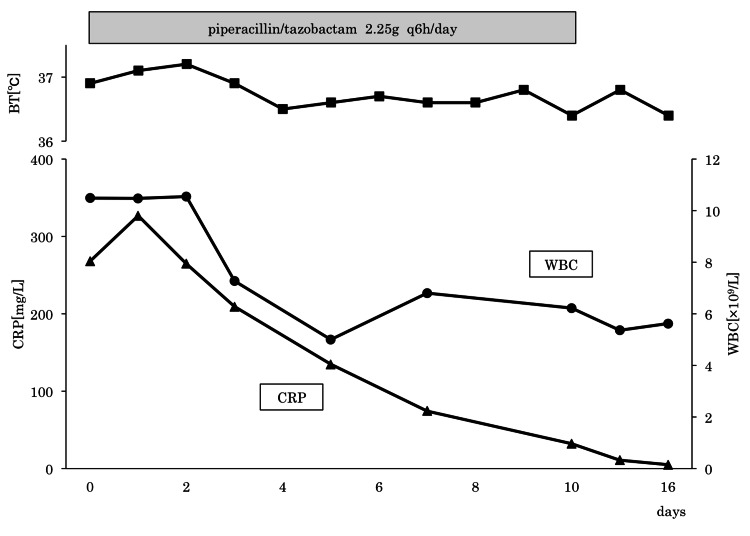
Clinical course after surgery for acute generalized peritonitis. BT: body temperature; CRP: C-reactive protein; WBC: white blood cell

Preoperative physical examination revealed a temperature of 36.3°C, heart rate 73/min, blood pressure 81/43 mmHg, and respiratory rate 14/min. During surgery, no suture failure or air leakage from the colonic anastomosis was observed. There were no perforation sites in the gastrojejunal anastomosis, small intestine, colon, or rectum. The patient had 1,700 mL of ascites fluid, which was washed out, and drains were placed under the right and left diaphragm and in the Douglas fossa. Postoperative antimicrobial therapy included piperacillin/tazobactam 2.25 g four times a day for 11 days. The patient resumed eating and was discharged with no recurrence (Figure 3).

**Figure 4:**
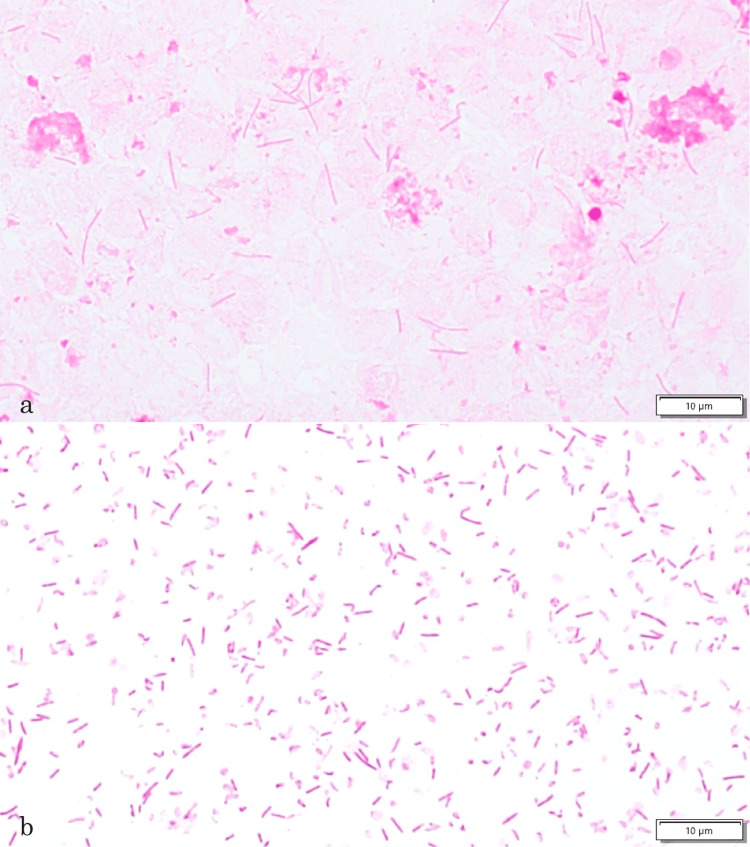
Gram stain findings of F. plautii blood cultures and colonies. (a) Blood cultures. (b) Colonies. Microscope magnification: ×1,000

Two sets of blood culture bottles were collected with venous blood prior to surgery for acute generalized peritonitis. Blood culture bottles BD BACTEC Plus Aerobic/F and BD BACTEC Plus Anaerobic/F (Becton, Dickinson and Company, Franklin Lakes, NJ, USA) were used, with 10 mL of blood collected in each. They were promptly transported to the laboratory and incubated for five days at 35°C in a BD BACTEC FX blood culture system. The two anaerobic bottles exhibited positive signals after 53 hours. Gram staining of the blood cultures revealed gram-negative rods, with spores in a subset of bacteria, indicating the presence of anaerobic bacteria (Figure 4).

**Figure 5:**
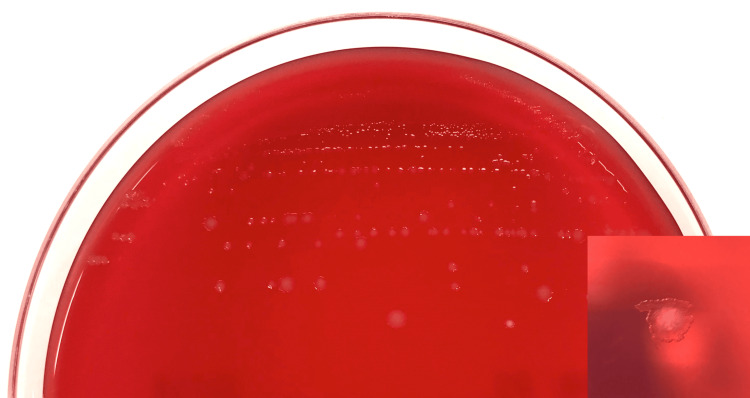
F. plautii colonies on Brucella HK agar (anaerobically incubated for 48 hours).

Gray colonies grew well on Brucella HK agar (RS) (Kyokuto Pharmaceutical Industrial Co., Ltd., Tokyo, Japan) at 35°C for 48 hours (Figure 5). The colonies displayed a thin, membrane-like growth pattern that spread as incubation time increased. After subculturing, colonies with a white center were observed, and bacteria with ubiquitous spores were present. Biochemical testing showed the bacteria were catalase-negative and oxidase-negative.

Identification using matrix-assisted laser desorption ionization-time of flight mass spectrometry (MALDI-TOF MS) with the MALDI Biotyper microflex LT/SH (Bruker Daltonics GmbH & Co. KG, Bremen, Germany) and MBT Compass Library Revision K (2022) confirmed *F. plautii* (score value: 2.32), with no other candidate species identified. In the ascites fluid, however, *Enterobacter cloacae*, *Enterococcus faecium*, *Eggerthella lenta*, *Lactobacillus* sp., and* Candida* sp. were detected, but *F. plautii* was not.

Antimicrobial susceptibility testing followed the methods outlined in the Clinical and Laboratory Standards Institute (CLSI) M100 for *Bacteroides* spp. and *Parabacteroides* spp. Brucella broth and RSMA1 (Shimadzu Diagnostics Corporation, Tokyo, Japan) were incubated under anaerobic conditions at 36°C ± 1°C for 48 hours, and the minimum inhibitory concentration (MIC) was determined (Table 1).

**Table 1:** Antimicrobial susceptibility results in this case. MIC: minimum inhibitory concentration

Antimicrobial	MIC (µg/mL)
Ampicillin/sulbactam	≤0.5/0.25
Piperacillin/tazobactam	≤2/4
Cefotaxime	≤4
Ceftriaxone	≤8
Cefoperazone/sulbactam	2/2
Cefmetazole	≤2
Flomoxef	≤1
Faropenem	≤0.25
Imipenem	≤0.25
Meropenem	≤0.25
Clarithromycin	>16
Azithromycin	>16
Clindamycin	>8
Levofloxacin	1
Moxifloxacin	≤1
Minocycline	≤2
Metronidazole	≤0.25

## Discussion

*F. plautii* is a 2-10-µm-long, straight or slightly curved, obligate anaerobic rod. The cells are gram-variable after staining, with motility and spore production being variable. The sporulation-specific gene *spo0A* is present. This bacterium can weakly ferment glucose, fructose, and ribose and is capable of cleaving quercetin and other flavonoids. It exhibits reduced susceptibility to vancomycin. The major metabolic end products in tryptone glucose yeast (TGY) extract broth are acetic and butyric acids. Nitrate is not reduced. The production of indole and H_2_S is variable. Gelatin and meat are not digested, and lecithinase is not produced. Colonies are minute, circular, convex, gray or white, smooth, and non-hemolytic on sheep blood agar [1].

As a component of the human gut microbiota, *F. plautii* has been studied in various fields. Kasai et al. reported that *F. plautii* was detected in stool specimens from more non-obese (0.22%) than obese (0.06%) individuals in a Japanese population, suggesting an association between gut microbiota and obesity [8]. Two genera in the family *Ruminococcaceae*, *Oscillibacter,* and *Flavonifractor* were significantly increased in the healthy group compared to the non-alcoholic fatty liver disease group [9]. The relative abundance of *F. plautii* in the feces of mice was higher in the catechin-rich green tea supplementation group. Furthermore, it was found that *F. plautii* alleviates mucosal damage by suppressing the overexpression of interleukin-17 [10]. Contrarily, a study from India, one of the countries with a very low incidence of colorectal cancer, reported that the presence of *F. plautii* was significantly associated with colorectal cancer fecal. The high abundance of *F. plautii*, a major flavonoid-degrading bacterium, was reported to be reasonably associated with high flavonoid degradation rates, which may minimize the potential beneficial effects and bioavailability of flavonoids in colorectal cancer samples [11].

*F. plautii* is part of the human microbiota, and there have been few reports of infection. It is unclear whether *F. plautii* is pathogenic to humans. Past cases are shown in Table 2 [2-7]. In previous case reports, infections occurred across various age groups, with 85.7% (6/7) of cases being male. There were no fatal cases. In 85.7% (6/7) of cases, the bacteria were presumed to have invaded through the intestinal tract. Therefore, diseases of the intestinal tract or fragility of the intestinal wall poses a risk for *F. plautii* bacteremia. Europe accounted for 71.4% (5/7) of the reports, possibly reflecting differences in gut microbiota due to dietary culture. Most previous reports involved severely immunocompromised patients, suggesting that the immunocompromised state may not eliminate this organism and may lead to bacteremia. The present case shows that bacteremia can occur even in mildly immunocompromised patients.

**Table 2:** List of case reports caused by F. plautii. CSF: cerebrospinal fluid; MALDI-TOF MS: matrix-assisted laser desorption ionization-time of flight mass spectrometry; AST: antimicrobial susceptibility testing; BMD: broth microdilution

Year	1991	2008	2018	2021	2022	2022	2024
Authors	Garre et al. [2]	Orlando et al. [3]	Berger et al. [4]	Karpat et al. [5]	Costescu Strachinaru et al. [6]	Wilton et al. [7]	The present case
Country	France	Italy	Germany	Austria	Belgium	Australia	Japan
Age(yr)/sex	35/M	33/M	69/M	24/F	45/M	62/M	83/M
Outcome	Survival	Survival	Survival	Survival	Survival	Survival	Survival
Primary diseases	Unknown	Kidney transplant	Prostate carcinoma	Beta-thalassemia	Severe burn	Hip dysplasia, atrial fibrillation	Diabetes mellitus, gastric cancer, hepatocellular carcinoma
Immunosuppression	Splenectomy	Tacrolimus, prednisone, mycophenolate sodium	Prostate carcinoma, chemotherapy	Splenectomy	Severe burn	-	Diabetes mellitus, gastric cancer, hepatocellular carcinoma
Source of infection	Dog bite	Bacterial translocation	Gangrenous cholecystitis	Infectious colitis	Bacterial translocation or catheters	Diarrhoeal illness	Generalized peritonitis
Samples	Blood, CSF	Blood, pleural effusion	Blood	Blood	Blood	Blood, hip joint synovial fluid	Blood
Antimicrobial therapy	Benzylpenicillin	Meropenem, vancomycin	Ceftriaxone, metronidazole	Meropenem, metronidazole	Amoxicillin/clavulanate	Ceftriaxone, metronidazole, amoxicillin/clavulanate	Piperacillin/tazobactam
Identification	Unknown	16S rRNA	16S rRNA, MALDI-TOF MS	Unknown	MALDI-TOF MS	MALDI-TOF MS	MALDI-TOF MS
AST	-	-	E-test	-	E-test	E-test	BMD
Gram stain	Negative	Variable	Positive	Unknown	Negative	Variable	Negative

The only device present when the blood culture was collected was a peripherally inserted central catheter (PICC). Potential sources of infection include bacterial translocation due to intestinal mucosal fragility or invasion from the intestines by generalized peritonitis. However, since no intestinal perforation was found during surgery and the bacterium was not detected in the ascites fluid, it is suggested that bacteremia was caused by bacterial translocation. Although the patient experienced strong abdominal pain and low blood pressure, septic shock did not occur. The patient was treated with intraperitoneal lavage, drainage, and administration of piperacillin/tazobactam, leading to recovery. There was approximately a two-month fasting period before the blood culture turned positive, during which the gut microbiota was in an abnormal state, possibly contributing to the development of bacteremia caused by this pathogen.

Foods rich in flavonoids, such as green tea, are common in Japan and may influence gut microbiota and related infections. Although the patient's green tea consumption during hospitalization could not be confirmed, there was an instance where the patient preferred hot green tea in tea bags over commercial tea. This species, like some *Clostridium* spp., may stain gram-negative, so caution should be exercised in reporting results due to the potential impact on initial antimicrobial therapy. Both blood culture bottles and gram staining of colonies showed variation in the length of the bacterium, which is characteristic of this species. Therefore, in patients with intestinal tract diseases, it is recommended to initiate treatment based on the timing of positive blood cultures and the type of bottles used, assuming the presence of this species. Only a very small number of spores were observed in the blood culture, but they formed ubiquitous spores in passaging cultures, indicating that they can form depending on culture conditions.

Ten strains of *F. plautii* are registered in the MBT Compass Library Revision K (2022), a MALDI-TOF MS library. As reported previously, *F. plautii* can be accurately identified by MALDI-TOF MS. In this and other cases, the MIC was low for many antimicrobials, but the MIC of clindamycin was high in this case, despite differences in antimicrobial sensitivity testing methods in previous case reports. Appropriate antimicrobial therapy for *F. plautii* has yet to be established, and further case accumulation is needed to understand the resistance mechanism and confirm the effectiveness of different antimicrobials.

## Conclusions

We described a case of bacteremia caused by *F. plautii* in a mildly immunocompromised patient with acute generalized peritonitis. The present case shows that bacteremia can occur even in mildly immunocompromised patients. In previous case reports, there were no fatal cases. In most cases, the bacteria were presumed to have invaded through the intestinal tract. Therefore, diseases of the intestinal tract or fragility of the intestinal wall poses a risk for *F. plautii* bacteremia.
